# Liver Transplantation for Primary Sclerosing Cholangitis (PSC) With or Without Inflammatory Bowel Disease (IBD)—A European Society of Organ Transplantation (ESOT) Consensus Statement

**DOI:** 10.3389/ti.2023.11729

**Published:** 2023-09-29

**Authors:** M. Carbone, A. Della Penna, C. Mazzarelli, E. De Martin, C. Villard, A. Bergquist, P. D. Line, J. M. Neuberger, S. Al-Shakhshir, P. J. Trivedi, U. Baumann, L. Cristoferi, J. Hov, B. Fischler, N. H. Hadzic, D. Debray, L. D’Antiga, N. Selzner, L. S. Belli, S. Nadalin

**Affiliations:** ^1^ Centre for Autoimmune Liver Diseases, Department of Medicina and Surgery, University of Milano-Bicocca, Milan, Italy; ^2^ European Reference Network on Hepatological Diseases (ERN RARE-LIVER), IRCCS San Gerardo dei Tintori, Monza, Italy; ^3^ Department of General, Visceral and Transplant Surgery, University Hospital Tübingen, Tübingen, Germany; ^4^ Hepatology and Gastroenterology Unit, ASST GOM Niguarda, Milan, Italy; ^5^ AP-HP Hôpital Paul-Brousse, Centre Hépato-Biliaire, Inserm Unité 1193, Université Paris-Saclay, FHU Hépatinov, Centre de Référence Maladies Inflammatoires des Voies Biliaires et Hépatites Auto-Immunes, Villejuif, France; ^6^ Karolinska Institute, Karolinska University Hospital, Stockholm, Sweden; ^7^ Norwegian PSC Research Center and Section of Gastroenterology, Department of Transplantation Medicine, Oslo University Hospital, Oslo, Norway; ^8^ Research Institute of Internal Medicine, Oslo University Hospital, Oslo, Norway; ^9^ Institute of Clinical Medicine, Faculty of Medicine, University of Oslo, Oslo, Norway; ^10^ Liver Unit, Queen Elizabeth Hospital, Birmingham, United Kingdom; ^11^ National Institute for Health and Care Research (NIHR) Birmingham Liver Biomedical Research Centre, Centre for Liver and Gastrointestinal Research, College of Medical and Dental Sciences, University of Birmingham, Birmingham, United Kingdom; ^12^ Division of Pediatric Gastroenterology, Hepatology and Liver Transplantation, Department of Pediatric Kidney, Liver and Metabolic Diseases, Hannover Medical School, Hannover, Germany; ^13^ Department of Pediatrics, Karolinska University Hospital, Karolinska Institutet, Stockholm, Sweden; ^14^ Department of Clinical Science, Intervention and Technology, Karolinska University Hospital, Karolinska Institutet, Stockholm, Sweden; ^15^ Paediatric Centre for Hepatology, Gastroenterology and Nutrition, King’s College, London, United Kingdom; ^16^ Unité d’Hépatologie Pédiatrique, Hôpital Necker-Enfants Malades, Centre de Référence Maladies Inflammatoires des Voies Biliaires et Hépatites Auto-Immunes, Filfoie, Paris, France; ^17^ Paediatric Hepatology, Gastroenterology and Transplantation, Hospital Papa Giovanni XXIII, Bergamo, Italy; ^18^ Multiorgan Transplant Program, University of Toronto, Toronto, ON, Canada

**Keywords:** liver transplantation, PSC, IBD, immunosuppression, retransplantation

## Abstract

Primary sclerosing cholangitis (PSC) is the classical hepatobiliary manifestation of inflammatory bowel disease (IBD) and a lead indication for liver transplantation (LT) in the western world. In this article, we present a Consensus Statement on LT practice, developed by a dedicated Guidelines’ Taskforce of the European Society of Organ Transplantation (ESOT). The overarching goal is to provide practical guidance on commonly debated topics, including indications and timing of LT, management of bile duct stenosis in patients on the transplant waiting list, technical aspects of transplantation, immunosuppressive strategies post-transplant, timing and extension of intestinal resection and futility criteria for re-transplantation.

## Introduction

Primary sclerosing cholangitis (PSC) is an immune-mediated disorder characterized by multi-focal bile duct strictures, progressive cholestatic disease, and heightened lifetime risks of cancer. Given the absence of definitive medical therapy, liver transplantation (LT) is the only life-extending intervention for patients with advanced disease. Disease recurs in approximately one-third of recipients, leading to graft loss and need for re-transplantation.

Although a rare disease, PSC accounts for 10%–15% of liver transplant activity in Europe and North America [1]. Alongside decompensated liver disease, transplantation may be considered for intractable cholestatic pruritus, deep and persistent jaundice and recurrent bacterial cholangitis. In some centers, high-grade biliary dysplasia and early cholangiocarcinoma are also accepted as indications [2].

The dominant clinical presentation of PSC is in association with gut inflammation, with 70%–80% of patients having inflammatory bowel disease (IBD). This relationship has driven several pathogenic hypotheses, in which enteric dysbiosis, dysregulated mucosal immune responses and altered bile acid metabolism are proposed to contribute [3, 4]. Additionally, there is a growing body of evidence that the clinical course of liver disease can be affected by IBD activity; and in turn, the natural history of colitis may be affected by that of PSC [4]. Indeed, data from large volume liver programmes suggest that ongoing intestinal inflammation, an intact colon and antibiotics might influence the clinical course of PSC, both before and after LT.

Although PSC, with and without IBD, is considered a standard indication for LT, many questions remain unanswered. To address these concerns, the European Society of Organ Transplantation (ESOT) convened a dedicated working group comprised of experts in PSC, IBD and LT. The overarching goal was to develop consensus recommendations relating to the:

1. Indication, timing and allocation rules of LT in patients With PSC, with and without IBD
2. Management of bile duct stenosis on the waiting list
3. Surgical aspects of LT
4. Immunosuppressive strategies in patients with PSC-IBD
5. Indication, Timing and extension of intestinal resection (i.e., colectomy) in patients with PSC-IBD
6. Futility criteria with regards re-transplantation

In so doing, the aforementioned topics were discussed in two virtual meetings and voted on during a face-to-face Consensus Conference that took place in person in Prague, 13–15 November 2022. The rationale, literature findings and recommendations from the Working Group on *PSC and IBD in LT setting* are presented in this article.

## Methods

The consensus development process was organized by a dedicated Guidelines’ Taskforce within ESOT, and its sections ELITA, EKITA, EPITA, ECTTA, ETHAP, Education Committee, YPT, Transplant International editorial board members and patient representatives. Detailed description of methodology is reported elsewhere [5].

Briefly, key issues related to the topic of *PSC and IBD in LT settings* were identified by the working group and specific clinical questions were formulated and agreed by the working group according to the PICO methodology (PICO = Population, Intervention, Comparator and Outcome). Following the definition of the PICOs, literature searches were developed by expert staff from the Centre for Evidence in Transplantation (CET) who have expertise in conducting systematic reviews and subsequently integrated, when needed, by the working group experts. A PRISMA flowchart describing the number of studies identified by the literature search and number of studies selected for inclusion in the consensus statement appears in Supplementary Figures S1A–L **.**

A summary of the selected studies addressing each key question is reported in Supplementary Tables S1–S5. The working group proposed recommendations based on the quality of evidence in relation to each question, using the GRADE approach: high quality rated as A, medium quality as B, low quality as C; very low quality as D. For evaluation of the quality of evidence according to GRADE [6], the following features were considered: study design, risk of bias, inconsistency, indirectness, imprecision, number of patients, effect, importance and publication bias (Table 1). In GRADE, recommendations can be strong or weak, in favor or against an intervention. Strong recommendations suggest that all or almost all persons would choose that intervention. Weak recommendations imply that there is likely to be an important variation in the decision that informed persons are likely to make (Table 2).

**TABLE 1:** Quality of evidence (GRADE).

GRADE	Definition
High	We are very confident that the true effect lies close to that of the estimate of the effect
Moderate	We are moderately confident in the effect estimate: the true effect is likely to be close to the estimate of the effect, but there is a possibility that it is substantially different
Low	Our confidence in the effect estimate is limited: the true effect may be substantially different from the estimate of the effect
Very Low	We have very little confidence in the effect estimate: the true effect is likely to be substantially different from the estimate of effect

**TABLE 2:** Strength of recommendation (GRADE).

Strength of recommendation	Definition
Strong	Desirable effect of intervention clearly outweigh undesirable effects, or clearly do not
Weak	Trade-offs are less certain, either because of low-quality evidence or because evidence suggests desirable and undesirable effects are closely balanced

The Delphi method was employed to reach a shared consensus among participants during the consensus conference. Complete information including the list of consensus conference workgroup domains (and topics noted below), the process regarding consensus conference participant selection, the development and refinement of consensus statements, and modified Delphi methodology including consensus polling, has been reported prior to the in-person conference held in Praque, Czech Republic, 13–15 November 2022 [5].

## Results

### 1. Indication, Timing and Allocation Rules of LT in Patients With PSC and IBD

**Question:** Is the MELD-based allocation scheme for organ from deceased donors a disadvantage in terms of waiting-list mortality for patients with PSC?
**Recommendation 1.1:** MELD should be used for prioritizing patients with PSC on the waiting list for LT. Although not disease-specific, it does not give a disadvantage in terms of waiting-list mortality compared to patients with other etiologies.
**Quality of Evidence:** low
**Strength of Recommendation:** weak for
**Consensus:** 88%
**Additional comment:** A PSC-specific model that captures the clinical burden of PSC more holistically should be developed.
**Recommendation 1.2:** For PSC-specific complications not reflected by the MELD score (e.g., recurrent cholangitis and/or pruritus), exception points should be considered.
**Quality of Evidence:** very low
**Strength of Recommendation:** weak for
**Consensus:** 92%

The MELD score is used to predict survival in the absence of transplantation. The score has been validated for many liver diseases. MELD (or one of its derivatives) is widely used to prioritize allocation of organs. However, as with any estimation of survival, application to an individual is less precise and allocation systems allow for this in a variety of ways such as awarding additional points for various indications (such as for liver cell cancer) or having a separate category for selected conditions where MELD score does not reflect prognosis or severely impaired quality of life [7].

For patients with PSC, we recommend that transplantation should be considered, irrespective of MELD score in some patients including those with intractable severe pruritus that makes the patient’s quality of life unacceptable, and/or recurrent bacterial cholangitis (at least two episodes requiring hospital admission within 1 year).

It should be noted that in many countries and under specific circumstances, individuals with PSC and documented, non-iatrogenic recurrent bacterial cholangitis, do receive additional MELD points and, thereby higher waiting list or allocation priority; even though some reports suggest that transplant candidates with PSC and recurrent cholangitis have no clear increase in mortality risk [8]. This raises the challenge of applying standardized listing procedures to the PSC population both in MELD-based and consensus-based transplant programs.

Several retrospective cohort studies across Europe and US report that PSC patients, while having significantly longer waiting time, have a lower time-dependent risk of death or removal from the waitlist in comparison with patients without PSC [8–10]. Of note, these comparisons were not age-matched (Supplementary Table S1).

Perhaps unsurprisingly, PSC patients with MELD Exception (ME) points have a significantly greater probability of undergoing LT than those without [10]. Moreover, the 90 days waiting list mortality in PSC patients is similar to that of individuals listed for chronic hepatitis C virus (HCV), and lower to that of alcohol-related liver disease (ALD) [11]. By contrast, PSC patients are less likely to be removed from transplant waiting lists in MELD-score based allocation programs, as compared to individuals with primary biliary cholangitis (PBC) and autoimmune hepatitis (AIH).

A German study analyzed the temporal effect and found no difference on the WL mortality in the pre-MELD versus the post-MELD era [12]. The mean time on the waiting list increased since introduction of MELD-based allocation from 1.6 to 2.3 years but this difference failed to reach statistical significance (*p* = 0.068). No improvement in means of short-term mortality could be shown in relation to alterations of allocation policy within the MELD era (Supplementary Table S1).

**Question:** Is LT for high-grade dysplasia (HGD) in suspicious strictures in patients with PSC an acceptable indication considering the risk of cancer recurrence?
**Recommendation 1.3:** Liver transplantation for individuals with PSC and high-grade biliary dysplasia, as confirmed by cytology or ductal histology, and the absence of other transplant indications, can be considered on an individualized basis, taking into account local resources and policies.
**Quality of Evidence:** very low
**Strength of Recommendation:** weak for
**Consensus:** 92%
**Additional comment:** A recall policy is recommended for those on the waiting list.

High grade dysplasia is a prelude to developing cholangiocarcinoma (CCA), and LT is routinely offered in this situation in some countries, specifically where (a) screening for dysplasia is systematically performed and (b) where the organ shortage is less marked [2, 13, 14]. However, the overall mortality of patients with HGD in explanted liver is similar to those with more benign histopathology (Supplementary Tables S2) [15]**.** Moreover, between 20% and 57% of patients who undergo LT for HGD, are not found to have cancer on explant histology, questioning the appropriateness of transplantation in patients with pre-neoplastic changes [2, 16].

Considering that 1) the risk of HGD development in PSC patients is difficult to quantify; and 2) the donor pool is limited in many countries, blanket recommendations of LT for HGD in PSC cannot be made.

### 2. Management of Patients on the Waiting List

**Question:** Is the empirical use of prophylactic, rotating antibiotics to prevent recurrent cholangitis in patients with PSC, compared to treatment on demand, a safe approach in LT candidates?

**Recommendation 2.1:** Rotating antibiotics may be considered to minimize the risks of recurrent cholangitis in selected patients. It is recommended that the use of rotating antibiotics follows biliary cultures and multidisciplinary review, due to the emergent risks of multidrug resistance (MDR).
**Quality of Evidence:** very low
**Strength of Recommendation:** weak for
**Consensus:** 90%

Positive bile cultures (even without clinical infection) are a common finding in patients with PSC. The analysis of bile obtained from liver explants of patients with PSC resulted in positive cultures in 21 out of 36 patients whereas in none of the 14 patients with PBC [17, 18]. Moreover, overt, clinically relevant bacterial cholangitis is a recognized complication, associated with biliary strictures and need for interventional procedures [1]. Biliary infections are often polymicrobial, with *Escherichia coli* being the most frequently identified pathogen. Other pathogens include gram-negative bacteria (e.g., *Klebsiella, Pseudomonas* and *Bacteroides*) and gram-positive bacteria (e.g., *Enterococci* and *Streptococci*) [18, 19]. The selection of antibiotic therapy is generally based on targeted organisms, local epidemiology, drug-resistance, renal and liver function, and severity of infection according to local policy [1]. In addition to antibiotic treatment, current guidelines recommend dilatation of clinically relevant strictures after multidisciplinary assessment [1, 20].

Recurrent episodes of bacterial cholangitis are a widely accepted indication for LT, even in the absence of cirrhosis. Whereas, the use of long-term rotational antibiotics to prevent recurrent bacterial cholangitis (spontaneous or after biliary endoscopy), in the absence of biliary cultures, is controversial; not least given that >25% of cirrhotic patients in Europe may harbor anti-microbial resistant bacteria [21]. Thus, empirical treatment with prophylactic long-term antibiotics should be avoided whenever possible due to a potential risk of furthering antimicrobial resistance. Therefore, this option should only be considered after multidisciplinary assessment in highly selected patients.

**Question:** In patients with PSC awaiting liver transplant, when is endoscopic biliary treatment, compared to observation only, justified for managing benign strictures?

**Recommendation 2.2:** ERCP can be considered in patients with clinically relevant strictures and severe symptoms that are likely to improve following biliary intervention. Balloon dilatation should be preferred versus stenting when treating biliary strictures endoscopically in PSC.
**Quality of Evidence:** moderate
**Strength of Recommendation:** weak for
**Consensus:** 100%

PSC patients with an indication for endoscopic intervention should be investigated initially with a high-quality MRI/MRCP [22] and discussed at a hepato-pancreato-biliary multidisciplinary meeting before ERCP is performed [1, 23]. Indications for ERCP in PSC include presence of clinically relevant strictures, sign/symptoms of obstructive cholestasis and/or bacterial cholangitis [1, 23]. There are no studies on the potential benefit or risk of endoscopic intervention in PSC patients on the transplant waiting list.

ERCP (especially with stenting) is a major risk factor for iatrogenic bacterial cholangitis, and peri-procedural antibiotics should be routinely used (EASL-ESGE guidelines) [23]. Decision-making about endoscopic intervention in PSC patients on the LT waiting list is complex and should be individualized.

In the pre-transplant setting, it may not always be obvious to determine whether an elevated or rising serum bilirubin value is caused by loss of liver synthetic function, other factors such as drug toxicity or bile duct strictures. A pragmatic approach to endoscopic treatment on the waiting list is to treat PSC patients with the aim of relieving symptoms, particularly in those with lower MELD scores and expected long waiting times. In individuals with advanced liver disease, ERCP should be reserved for the treatment of unacceptable symptoms, when the benefit is thought to outweigh risk [24]. In waitlisted patients, who have previously been treated with repeated dilatations or stenting, further treatment during the waiting time may be justified following MDT discussion with their transplant center.

Endoscopic intervention of biliary strictures is most useful for well-defined high-grade strictures in the larger bile ducts [23]. Balloon dilatation is treatment of choice when treating biliary strictures endoscopically in PSC, and stenting for benign disease should be avoided due heightened risks of complications without additional benefit [25, 26]. Needless to say, it is always advisable that an experienced biliary endoscopist should perform ERCP in this delicate setting.

### 3. Technical Issues and Graft Selection

**Question:** In liver transplant recipients with PSC, is duct-to-duct anastomosis preferred over hepaticojejunostomy as the type of biliary anastomosis?

**Recommendation 3.1:** The choice of biliary anastomosis is left to operator discretion. However, duct-to-duct anastomosis is recommended as the reconstructive technique of choice whenever technically feasible.
**Quality of Evidence:** moderate
**Strength of Recommendation:** strong for
**Consensus:** 100%

There is a lack of literary consensus on the ideal biliary reconstruction technique in LT of patients with PSC. Historically, hepaticojejunostomy (HJ) was preferred owing to the perceived risk of complications (including recurrent or *de novo* CCA) on the biliary anastomosis in a disease that often involves the extra-hepatic bile ducts. However, the incidence of anastomotic strictures (AS) is similar between HJ and duct-to-duct anastomosis (DD), albeit with a reduced risk of ascending cholangitis with the latter [27]. Moreover, the incidence of cholangiocarcinoma in the remnant BD system, and the 1 year incidence of biliary leaks and anastomotic strictures, does not appear to be different between patient groups stratified by anastomosis type [28]. Perhaps most striking, acute cholangitis episodes within the first year and non-AS (NAS) beyond the first post-transplant year, appear to be more frequent in the HJ group [29–32].

Apart from the above outlined outcomes, duct-to-duct reconstruction confers certain advantages as compared to HJ. It maintains a more ‘normal’ bile duct anatomy, preserves sphincter of Oddi function, and provides easier endoscopic access to the biliary tree if and when needed. This is of particular relevance in PSC, since 10%–30% of the patients may develop recurrent disease during the first 5–10 post-transplant years [33].

**Question:** Is the use of extended criteria donors (ECD) acceptable in liver transplantation for PSC?
**Recommendation 3.2:** Extended criteria liver grafts should be used with caution, considering the risk-benefit balance, given heightened risks of post-transplant biliary complications.
**Quality of Evidence:** weak
**Strength of Recommendation:** strong for
**Consensus:** 80%

Extended criteria grafts, in particular those with high grade steatosis (i.e., >30% macro-steatosis) and grafts from older donors (i.e., >55 years old), represent risk factor for post-transplant complications, including recurrent biliary disease [33–35]. The use of livers donated after circulatory death (DCD) has also been associated with heightened risks of ischemic type biliary strictures [28, 36]. The number of studies is low, and existing reports are heterogeneous in terms of graft types studied and classifications applied. Within DCD groupings, there are differences in procurement protocol, graft quality, and the risks of ischemic damage to bile ducts depending on whether normothermic regional perfusion was utilized. Furthermore, the use of machine perfusion after organ retrieval has been shown to reduce the incidence of NAS, but no study has reported specific outcomes in LT for PSC [37].

### 4. Immunosuppressive Strategies

**Questions:** What is the optimal immunosuppression regimen for adult patients transplanted for PSC?

**Recommendation 4.1:** The optimal immunosuppression regimen must be tailored to the needs of the individual and depends on many factors, in particular the heightened risks of rejection in PSC.
**Quality of Evidence:** high
**Strength of Recommendation:** strong for
**Consensus:** 100%
**Recommendation 4.2:** As acute rejection is associated with PSC recurrence, it is recommended that patients transplanted for PSC should start on a triple-immunosuppression regimen based on tacrolimus, an anti-proliferative agent and corticosteroids.
As acute cellular rejection may develop also late after transplantation, consideration should be given to maintaining such patients on dual or triple therapy long term.
**Quality of Evidence:** moderate
**Strength of Recommendation:** weak for
**Consensus:** 100%
**Recommendation 4.3:** We recommend against empirical protocol switching from a tacrolimus-to cyclosporin-based regimen. In transplantation for immune-mediated liver diseases like PSC, the merits of cyclosporin vs. tacrolimus use must be counterbalanced with risks of allograft rejection and acute kidney injury.
**Quality of Evidence:** low
**Strength of Recommendation:** weak for
**Consensus:** 100%

Despite a wide armamentarium of available immunosuppressive therapy, there is no evidence-based accepted immunosuppressive strategy in PSC recipients [38, 39]. This should ideally be tailored to the complication more often encountered in PSC such as early and late acute rejection and recurrent disease [40].

There are many studies evaluating the impact of different immunosuppressive regimens on a variety of outcomes, although very few are randomized, prospective and long term. Additionally, very few studies take into account variations in dose or cumulative levels of medications, and changes in regimen over time. Therefore, any conclusions drawn from studies looking at outcomes related to immunosuppression will need to be cautious and limited.

Cyclosporin (CyA) has shown a marginal benefit on recurrent PSC (rPSC) compared to tacrolimus (Tac). However, this has been attributed to a “era” effect rather than a pharmacological one [33]. Considering that early and late acute rejection has been widely associated with rPSC [41], the priority in patients transplanted for PSC should be to avoid early acute rejection through a triple-immunosuppression regimen (ideally Tac-based) and late acute rejection on dual therapy [42, 43].

The inferiority of azathioprine (AZT) over mycophenolate mofetil (MMF) on overall survival has been suggested in some studies [44], although not confirmed in follow-up studies [40, 45].

The impact of immunosuppressive regimen on the IBD activity add another layer of complexity to manage PSC patients and this will be discussed in the next paragraph.

### 5. Management of IBD Before and After Liver Transplant for PSC

**Question:** What is the optimum (safety/efficacy) therapeutic approach for maintaining remission in IBD associated with PSC pre-, peri- and post-LT?

**Recommendation** **5.1A:** In patients on antimetabolites, Azathioprine is favoured over mycophenolate post-LT, as maintenance therapy for PSC-associated colitis
**Quality of Evidence:** moderate
**Strength of Recommendation:** strong for
**Consensus:** 93%
**5.1B:** Anti-TNFα therapy should be used with caution in patients with a history of bacterial acute cholangitis
**Quality of Evidence:** moderate
**Strength of Recommendation:** strong for
**Consensus:** 100%
**5.1C:** Anti-TNFα therapy may be administered post-LT alongside CNI, provided that antimetabolites (AZT/MMF) have been stopped.
**Quality of Evidence:** Very low;
**Strength of Recommendation:** strong for
**Consensus:** 100%

No randomized controlled clinical trials, specifically to attenuate IBD activity in PSC have been found. Thus, clinical data are limited to largely retrospective case series and observational cohort studies. Persistent inflammatory activity pre-transplant can affect IBD behavior post LT, with a 3-fold greater risk of acute colitis “flares.” Among transplant recipients, the cumulative probability of deterioration in colitis activity at 10 years is estimated to range between 25.5% and 40%, despite ongoing use of anti-rejection/immunosuppression [46–48]. All efforts to attain mucosal healing in PSC should be pursued, particularly for patients with evidence of progressive liver disease over time that will ultimately require re-LT. This is particularly relevant given that a) PSC is invariably a progressive liver disease, b) LT is the only life-extending intervention for PSC patients, and c) ongoing IBD activity is associated with a heightened risk of peri- and post-transplant complications including hepatic artery thrombosis, rPSC and overall rates of graft loss.

European and American guidelines [1, 23] recommend that 5-ASAs may be used in the pre- and post-transplantation period for the induction and maintenance of remission in IBD associated with PSC. Corticosteroids may be used for the induction of remission in PSC-associated IBD, and as a bridge to escalating treatment.

Thiopurines, principally azathioprine (AZT), can be used to maintain remission from IBD pre- and post-transplantation, and does not adversely affect post-operative outcomes nor the risks of PSC-associated cancers [48–51]. Whilst differences in ciclosporin vs. tacrolimus have been suggested, they may reflect an era effect in transplant practice, which is less apparent for azathioprine vs. mycophenolate treatment paradigms.

Given its comparative safety profile and limited off-target effects, retrospective studies favoring the anti-a4b7 agent, vedolizumab, have also been assessed [52]. In a multicenter cohort of 16 and 14 PSC patients with Crohn’s disease and UC, respectively, with a median follow-up of 9 months, clinical remission was evident in 29% (PSC-UC) and 55% (PSC-Crohn’s disease) of patients following 30 weeks of therapy. A systematic review of vedolizumab use among liver transplant recipients (eight studies) indicates greater response rates than pre-transplant studies, with 20/27 patients reporting clinical improvement over a mean follow-up of 5–20 months. However, seven/31 patients experienced an infectious event after a mean-time vedolizumab exposure of 11.4 months [52].

The two most commonly used anti-TNFα agents are infliximab and adalimumab. Safety outcomes in relation to biologics mostly concern opportunistic infections, particularly when used in combination with other immunosuppressive agents [46, 53–55]. Pre-transplant data also indicates a sevenfold heightened risk of developing acute cholangitis with anti-TNFα agents (compared to no anti-TNFα treatment) [56]. Pragmatically, there is rationale from a safety point of view to minimize immunosuppressive burden among transplant recipients commencing anti-TNFα therapy, whilst balancing the risks of allograft rejection and recurrent disease. For instance, this may include cessation of corticosteroids and antimetabolites agents in patients who are being treated with calcineurin inhibitors and anti-TNFα therapy simultaneously. At present, there is no published data studying the safety and efficacy of newer biological agents post-transplant such as those directed toward Janus Kinase and/or IL12/23.

Several retrospective studies have shown the use of tacrolimus was associated with progression of IBD and increased risk of *de novo* IBD post-transplant [57]. In the absence of robust evidence, we cannot provide any recommendation on the CNI regimen concerning IBD activity.

**Question:** Which individuals with PSC-associated colitis should undergo (sub/total) colectomy?
**Recommendation 5.2:** We recommend (sub/total) colectomy in the following situations, among patients who are fit for surgery:
**5.2A)** Resectable colorectal cancer/neoplasia
**Quality of Evidence:** high
**Strength of Recommendation:** strong
**Consensus:** 100%
**5.2B)** High grade colonic dysplasia
**Quality of Evidence:** high
**Strength of Recommendation:** strong
**Consensus:** 100%
**5.2C)** Low grade dysplastic lesions with high-risk features (e.g., flat/invisible lesions) or multi-focal (synchronous or metachronous) low-grade dysplastic lesions
**Quality of Evidence:** low
**Strength of Recommendation:** strong
**Consensus:** 93%
**5.2D)** Fulminant colitis
**Quality of Evidence:** high
**Strength of Recommendation:** strong
**Consensus:** 100%
**5.2E)** Active colitis-refractory to medical therapy
**Quality of Evidence:** high
**Strength of Recommendation:** strong
**Consensus:** 100%
**5.2F)** Evidence of progressive liver disease (albeit well-compensated) and persistent colitis despite 5ASAs, AZTs (thiopurines) and a single biological agent
**Quality of Evidence:** low
**Strength of Recommendation:** strong
**Consensus:** 93%

Patients with PSC-associated ulcerative colitis harbor heightened lifetime risks of colonic dysplasia and colorectal cancer (CRC), as compared to their age- and sex-matched counterparts with UC alone, and against the general population [58–62]. Moreover, the majority of cancers tend to develop in the proximal colon [63, 64]. Of note, colorectal cancer is among the leading causes of death in patients with PSC-IBD [58, 59]. Risks persist after LT [65, 66], with an estimated CRC incidence rate of 5.8–13.5 per 1,000 patient years [47].

The risk of progression of low-grade dysplasia (LGD) in PSC-associated colitis is not fully quantified. It is likely that progression occurs within the first year of initial detection of LGD, and that flat lesions possess the greatest risk [67], similar to the background IBD population [68]. Thus, international guidelines prompt consideration of surgery (colectomy) with curative intent in patients with colitis and flat LGD, any degree of HGD, and in those with overt neoplasia that is deemed resectable provided patient fitness/comorbidities allow [69, 70].

In addition to CRC risk, colitis activity refractory to medical treatment is the commonest indication for colonic resection in PSC patients [58, 71–73]. It is generally accepted that the definition of fulminant colitis is similar in PSC-associated colitis and in UC alone—the indication for colectomy herein is rarely debated [74, 75]. However, for patients with steroid-dependent or steroid refractory chronic colitis, there is lack of consensus as to what stage colectomy should be performed.

As PSC is an invariably progressive disease, with LT being the only life-extending intervention, there is premise for adopting a lower threshold with regards colonic resection in these patients compared to those with IBD alone. In fact, colitis refractory to single (maximum two) biological agents warrants referral to (or at least discussion with) colorectal surgery. This is relevant given 1) the risks of colonic resection in patients with cirrhosis and portal hypertension, 2) the risks of multivisceral surgery (colectomy at the time of LT), and 3) the impact of persistent colitis activity on peri-/post-transplant complications (e.g., hepatic artery thrombosis) [76, 77].

**Question:** What is the optimal timing of (sub/total) colectomy for non-oncology indication?
**Recommendation 5.3:** We recommend that subtotal colectomy for non-oncology indication is performed for patients who have an indication (see recommendations 5.2E above) prior to the onset of advanced liver disease. This is to specifically minimize future risks of native liver decompensation (in patients who develop cirrhosis), post-LT recurrent disease, and graft loss post-LT
**Quality of Evidence:** moderate
**Strength of Recommendation:** strong for
**Consensus:** 93%

There are no comparative data stratifying the benefits vs. risks of colectomy according to the extent of ductal disease involvement, liver disease stage or the risk of disease recurrence. Nevertheless, data from chronic liver disease cohorts (including patients with PSC) highlight significant peri- and post-operative mortality following colectomy among patients with advanced liver disease compared to those with earlier stages (detailed in later sections, below) [78, 79].

Early studies showed that patients with more aggressive PSC liver disease requiring LT had a milder clinical course of IBD, with less need of colectomy pre-transplant [80, 81]. Reciprocally, patients in need of colectomy due to severe colitis can manifest less severe features of PSC liver disease [82].

A systematic review and metanalysis of seven studies post-colectomy, estimated a 2.11% per year overall mortality risk among patients with PSC, unstratified for indication and severity of liver disease [83]. Two studies directly compared colectomy vs. no colectomy groups and showed no difference in overall mortality across all evaluated time points (15.3% vs. 11.8% at 3 years in one study; and 17.4% vs. 20.4% over a median follow-up time of 5.9 years in another) [84, 85]. However, risk-stratified survival analysis of matched patient groups, who met indications for colectomy and underwent resection, versus those who met indications but did not have surgery, has not been performed.

The impact of colectomy on PSC-prognosis has been reported from a study of 45 PSC-IBD patients in whom colectomy did not affect liver function [84]. Other small studies, not primarily designed to investigate the effect of colectomy on PSC-prognosis, concluded that colectomy had no impact on liver-related prognosis [86–88]. However, emerging data from the pediatric literature indicates that late-onset colitis (>6 months following PSC diagnosis) is associated with higher rates of clinically significant portal hypertension [5/11 (45%) vs. 3/26 (12%); *p* = 0.007] and LT [5/11 (45%) vs. 2/26 (8%]; *p* = 0.02) over a median follow-up duration of 54 months [89]. Moreover, nationwide data from Sweden (*N* = 2,594) shows that very early colectomy (prior to, or close to the onset of PSC) is associated with a lower risk of LT/death (hazard ratio: 0.71, 0.53–0.95), with a 5 and 10 years incidence of 14.0% and 25.5%, respectively. This was as compared to 20.7% and 33.0% among those without colectomy [85].

At present, there are no data to support routine pre-vs. post-transplant colectomy timings, with regards the safety and efficacy of the colonic resection procedure itself. However, patients with advanced liver disease (i.e., cirrhosis) carry a greater risk of morbidity and mortality following any operation.

Presently, there are no data to support the empirical use of transjugular intrahepatic portosystemic shunts (TIPSS) to mitigate peri-/post-operative risk among patients with cirrhosis. In fact, data from a single retrospective study showed a heightened risk of complications among PSC patients undergoing TIPSS prior to colectomy (greater proportion with wound infections and wound dehiscence, longer hospital stays: 5 days vs. 8 days, and higher readmission rates) [90].

There is limited literature available comparing outcomes related to pre-vs. post-liver transplant colectomy, or to suggest the optimal timing of colonic resection post-transplant. Poritz et al. suggest that patients with PSC who require colectomy may undergo simultaneous LT and total abdominal colectomy [71], and other investigators have described this approach across their own respective practices [33, 57, 65].

**Question:** How does the type of colectomy (i.e., restorative vs. non-restorative/ileal pouch-anal anastomosis vs. ileostomy alone) affect liver outcomes?
**Recommendation 5.4:** When colectomy is indicated, it is imperative to provide patients with comprehensive counseling regarding their choice of restorative surgery. Patients should be empowered to weigh the benefits of avoiding a stoma against the increased risks associated with ileal pouch-anal anastomosis, including graft loss, non-anastomotic biliary stricture, and hepatic artery thrombosis. Additionally, patients should be informed about potential implications on their quality of life, as well as the heightened risks of acute pouchitis and pouch failure.
**Quality of Evidence:** moderate
**Strength of Recommendation:** strong for
**Consensus:** 86%

Data linking the type of colonic resection and liver-related outcomes are largely descriptive, with few comparative studies. Whilst the failure rate of ileal pouch-anal anastomosis (IPAA) and ileo-rectal anastomosis (IRA) in PSC-IBD may be no different to that of UC alone [91], the cumulative incidence of acute pouchitis (31% vs. 14% at 10 years), overall pouch related dysfunction (Oresland score: 7.7 vs. 5.4) and poor nocturnal pouch function is significantly greater in patients with PSC [92, 93]. Additionally, patients with large duct PSC and an IPAA exhibit a markedly lower quality of life compared to individuals with UC alone and an IPAA.

Epidemiological data from the Netherlands show how patients that undergo colectomy and retain a permanent ileostomy are at a significantly lower risk of needing a liver transplant/dying over time [HR 0.47 (0.24–0.93)] compared to patients without colectomy. In turn, sensitivity analysis shows no beneficial effect for colectomy with a pouch (HR 0.95, 0.62–1.44) [94] (No full publication, data in abstract version).

Very early studies suggest that approximately 50% of patients who undergo colonic resection may be at risk of developing ileostomal varices [95]. However, contemporary data are lacking, and there is no validating evidence to indicate such high risks in non-cirrhotic PSC.

In the post-transplant setting, there appears to be a significant difference in the incidence of graft loss between patient groups with an IPAA, end-ileostomy and those without a colectomy, with data from one large-volume center (*n* = 240) showing 10 years graft survival rates of 70%, 95% and 88%, respectively, *p* = 0.038 [96]. These differences were seen to persist on sub-analysis of patients undergoing colonic resection pre-transplant. With regards graft-related complications, the rate of hepatic artery thrombosis was also elevated in the IPAA group by more than 4-fold compared to the end ileostomy group; whereas end-ileostomy appeared to have a protective effect including against non-anastomotic biliary stricturing disease.

In conclusion, colectomy and retention of an end ileostomy is associated with lower risks of: 1) disease progression in the native liver compared to those having a restorative IPAA; 2) graft loss; 3) non-anastomotic biliary stricturing; 4)hepatic artery thrombosis compared to IPAA and no colectomy. Patients undergoing colectomy should be counselled about the risks of IPAA with regards to quality of life, acute pouchitis, pouch failure and liver/graft-related outcomes.

### 6. Post Transplant Course

**Question:** Are there criteria of futility for re-LT in case of rPSC?

**Recommendation 6.1:** Patients with recurrent PSC and graft failure can be offered re-transplant, if expected patient’s survival is more than 50% at 5 years, taking in consideration local waiting list mortality and surgical issues.
**Quality of Evidence:** very low
**Strength of Recommendation:** strong for
**Consensus:** 100%

Re-transplantation in rPSC is controversial, because of the historical lower patient and graft survival rates compared with primary transplantation, due to surgical challenges and septic complications. This raises ethical concerns on utility and equity in the use of a scarce resource (liver organ) for a disease that will tend to recur, sometimes more than once.

Several studies have explored the impact of rPSC on patient survival showing conflicting results [97–103]. This might be related to the different study design and study limitations, e.g., small sample size, short follow-up time, single vs. combined endpoints used, selection bias in patient selection. In some studies, the evidence of recurrence was not included as time-varying covariate, therefore disregarding the impact of survived time until rPSC development on the overall.

A recent analysis of the ELTR data, on 1,549 patients undergoing LT for PSC over a period of 35 years (1980–2015), reported graft survival (including re-transplants) at 1, 5, 10 and 20 years of 80%, 70%, 60% and 41%, respectively. This survival rate is far superior to the expectation of at least 50% at 5 years that has been proposed by the transplant community as a minimum threshold to avoid futility [104]. The rate of rPSC was 17%, including re-transplants, after a median of 5.1 years. Authors reported a negative impact of rPSC on patient survival (HR = 2.3) independent of other transplant related co-variates. Patients with rPSC underwent significantly more re-transplants than those without rPSC (OR 3.6). Notably, patients affected by rPSC did benefit from re-transplantation, showing a patient survival similar to that of patients without rPSC but re-transplanted for other causes. Moreover, in patients with and without rPSC, 5 years graft survival for second graft was noted to be 77% vs. 79%, with no difference in patient survival.

Similar results come from the analysis of the UNOS/OPTN database of 5,080 PSC patients who received LT in the US [105]. Recipients of re-LT for rPSC were more likely to be in the ICU or on mechanical ventilation at LT, and they also had a greater degree of hepatic and renal dysfunction. However, their outcomes were similar at 5 years. Furthermore, the majority of wait-list deaths from rPSC occurred within 6 months, highlighting the risk of not receiving re-LT. Putting together these data, considering the favorable post-re-LT outcomes and the high proportion of waitlist mortalities occurring soon after relisting, support the consideration of re-LT in patients with rPSC.

Patients who undergo a second liver transplant for rPSC have similar graft and patient survival than those transplanted for other causes.

An important caveat to this statement though is that the patients included in this analysis were likely highly selected to undergo re-LT for their favorable pre-LT characteristics

While these data are based on the largest multicenter study on rPSC post-transplant, granular patient data, such as imaging and biopsy, were only available for a minority (approximately one-third of all the transplant center included in the ELTR and not available in the UNOS/OPTN database). Conclusions are limited by several factors inherent with retrospective review of a large administrative database, including missing, incomplete, or potentially inaccurate data.

At the time being, based on a pure needs and outcomes standpoint, it seems reasonable to continue offering re-transplant to patients with rPSC until further prospective studies demonstrate otherwise.

## Liver Transplantation for Children With PSC and IBD

Although PSC in adults shares many features with the same condition in children, some clinicopathological features may differ at pediatric age, including rate of progression, severity of pruritus, or development of biliary strictures and malignancies. In pediatrics, the diagnosis typically occurs in the second decade, and most children do not require a LT in childhood. Alongside, the risk of cholangiocarcinoma is very low before 18 years of age. The pediatric studies on PSC are scarce and their quality of evidence remains limited [106–109]. Furthermore, the balance between the existing data and clinical impact of recommended interventions could vary at different ages. For instance, re-transplantation is usually not controversial for children with recurrent PSC in a failing graft. Similarly, suggesting colectomy with a permanent ileal-pouch has very different social implications in children compared to the adults. For these reasons the recommendations produced for the adult patients have been largely supported by the pediatric co-authors when applicable but the guidance from this document should be tailored to the individual patients following multidisciplinary input and discussion.

## Summary and Next Steps

No therapies have proved to cure PSC or slow down disease progression and most patients ultimately require LT. Transplantation faces several challenges in PSC, from the fairness of the extra-MELD indications, the donor selection and the technical issues, to the disease recurrence with risk of graft loss. The association between IBD and recurrence, underscores the interplay between the bowel and the liver in PSC patients.

The systematic literature review undertaken for these recommendations, highlighted for many of the topics a low-quality level of evidence and statements were often based on clinical expertise. Prospective clinical studies on the debated topics are urgently needed.

## Figures and Tables

**Table:** 

**AGA**	American Gastroenterological Association
**AIH**	autoimmune hepatitis
**ALD**	alcohol-related liver disease
**AS**	anastomotic strictures
**ASA**	aminosalicylic acid
**AZT**	azathioprine
**CET**	Centre for Evidence in Transplantation
**CCA**	cholangio-carcinoma
**CYA**	cyclosporin
**DD**	duct-to-duct anastomosis
**EASL**	European Association for the Study of the Liver
**ERCP**	endoscopic retrograde cholangiopancreaticography
**ESOT**	European Society of Organ Transplantation
**ELITA**	European Liver and Intestine Transplant Association
**EKITA**	European Kidney Transplant Association
**EPITA**	European Pancreas and Islet Transplant Association
**ECD**	extended criteria donors
**ECTTA**	European Cardio Thoracic Transplant Association
**ETHAP**	European Transplant Allied Healthcare Professionals
**HCV**	Hepatitis C virus
**HD**	hepaticojejunostomy
**HGD**	high grade dysplasia
**IBD**	Inflammatory Bowel Disease
**IPAA**	ileal pouch-anal anastomosis
**LT**	liver transplantation
**MDR**	multidrug resistance
**ME**	MELD Exception
**MELD**	model for end-stage liver disease
**MMF**	mycophenolate mofetil
**MRC**	magnetic resonance cholangiography
**MRCP**	magnetic resonance cholangiopancreatography
**MRE**	magnetic resonance elastography
**NAS**	non-anastomotic strictures
**OPTN**	Organ Procurement and Transplantation Network
**PICO**	Population/Intervention/Comparison/Outcome
**PBC**	primary biliary cholangitis
**PSC**	Primary Sclerosing Cholangitis
**RCT**	randomised-controlled trial
**rPSC**	recurrent primary sclerosing cholangitis
**TAC**	tacrolimus
**TIPS**	transjugular intrahepatic portosystemic shunt
**TNF**	tumour necrosing factor
**UC**	ulcerative colitis
**UDCA**	ursodeoxycholic acid
**UNOS**	United Network for Organ Sharing

## Data Availability

The original contributions presented in the study are included in the article/Supplementary Material, further inquiries can be directed to the corresponding author.
