# Supplementary material for: Liver Transplantation for Primary Sclerosing Cholangitis (PSC) With or Without Inflammatory Bowel Disease (IBD)—A European Society of Organ Transplantation (ESOT) Consensus Statement
Source: Transpl Int. 2023 Sep 29;36:11729. doi: 10.3389/ti.2023.11729 (PMC10570452; doi:10.3389/ti.2023.11729)
Supplement: Supplementary file 1 [file DataSheet1.PDF]

Supplementary material

Liver transplantation for Primary Sclerosing Cholangitis (PSC) and Inflammatory Bowel Disease (IBD) - a European Society of Organ Transplantation (ESOT) Consensus Statement

Carbone M [1], Della Penna A [2], Mazzarelli C [3], De Martin E [4], Villard C [5], Bergquist A [5], Line PD [6], Neuberger JM [7], Al-Shakhshir S [8], Trivedi PJ [8], Baumann U [9], Cristoferi L [1], Hov J [6], Fischler B [10], Hadzic N [11], Debray D [12], D'Antiga L [13], Selzner N [14], Belli L [3], Nadalin S [2]
on behalf of the ESOT Guidelines Taskforce

Supplementary figure 1 (a-l). PRISMA flowchart describing the number of studies identified by the literature search and number of those selected for inclusion in the consensus statements

a)

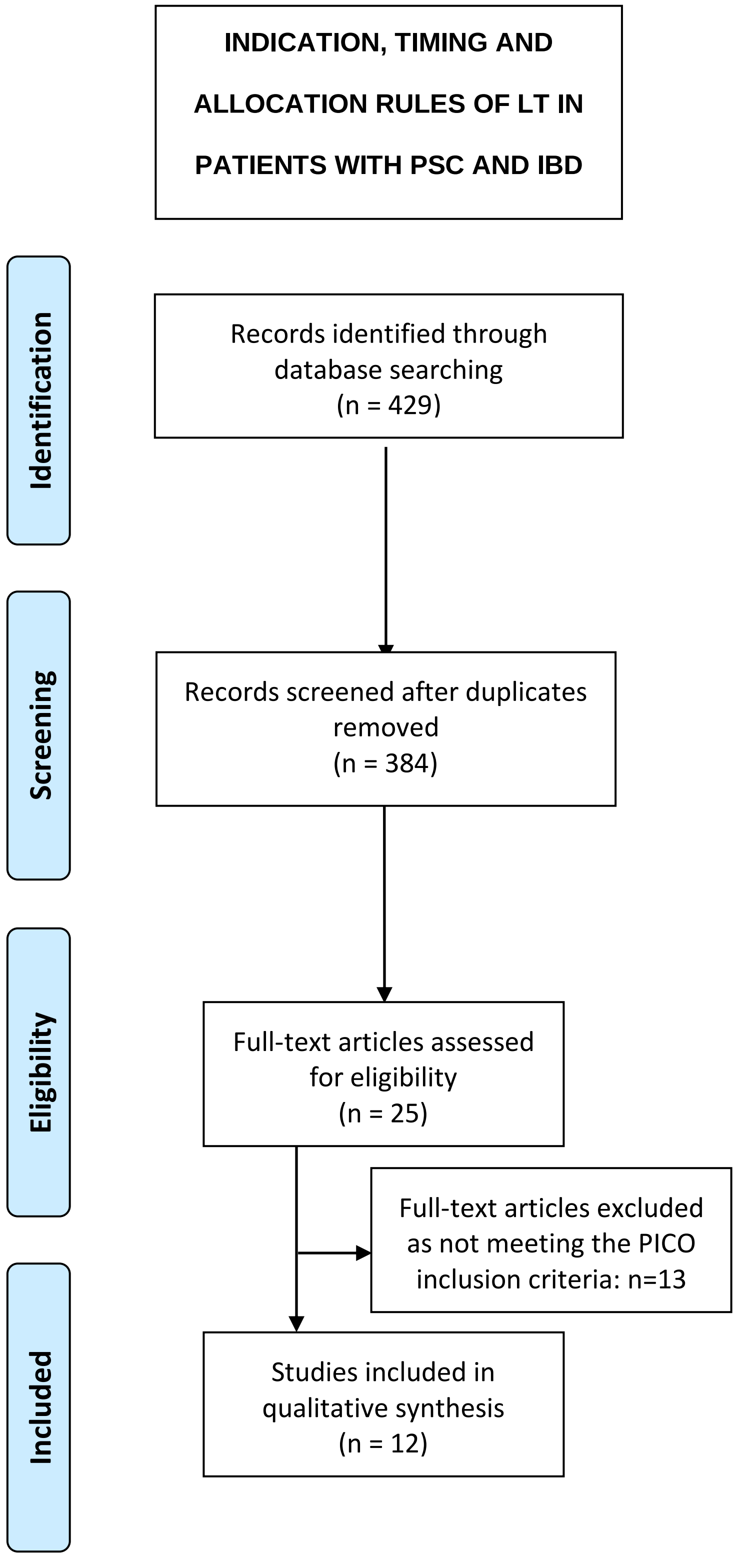

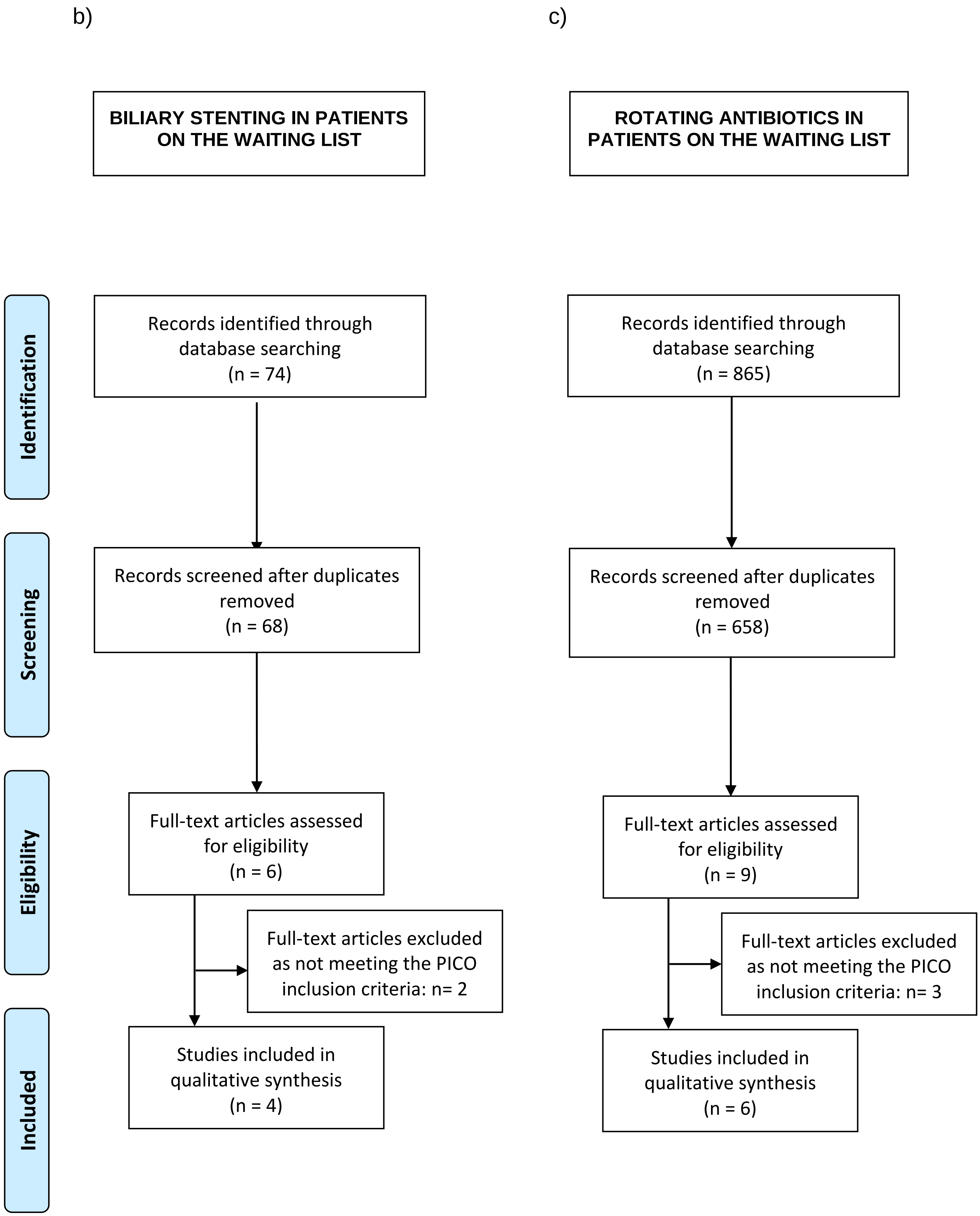
b)
c)
BILIARY STENTING IN PATIENTS ON THE WAITING LIST
ROTATING ANTIBIOTICS IN PATIENTS ON THE WAITING LIST
Identification
Screening
Eligibility
Included
Records identified through database searching (n = 74)
Records screened after duplicates removed (n = 68)
Full-text articles assessed for eligibility (n = 6)
Full-text articles excluded as not meeting the PICO inclusion criteria: n= 2
Studies included in qualitative synthesis (n = 4)
Records identified through database searching (n = 865)
Records screened after duplicates removed (n = 658)
Full-text articles assessed for eligibility (n = 9)
Full-text articles excluded as not meeting the PICO inclusion criteria: n= 3
Studies included in qualitative synthesis (n = 6)

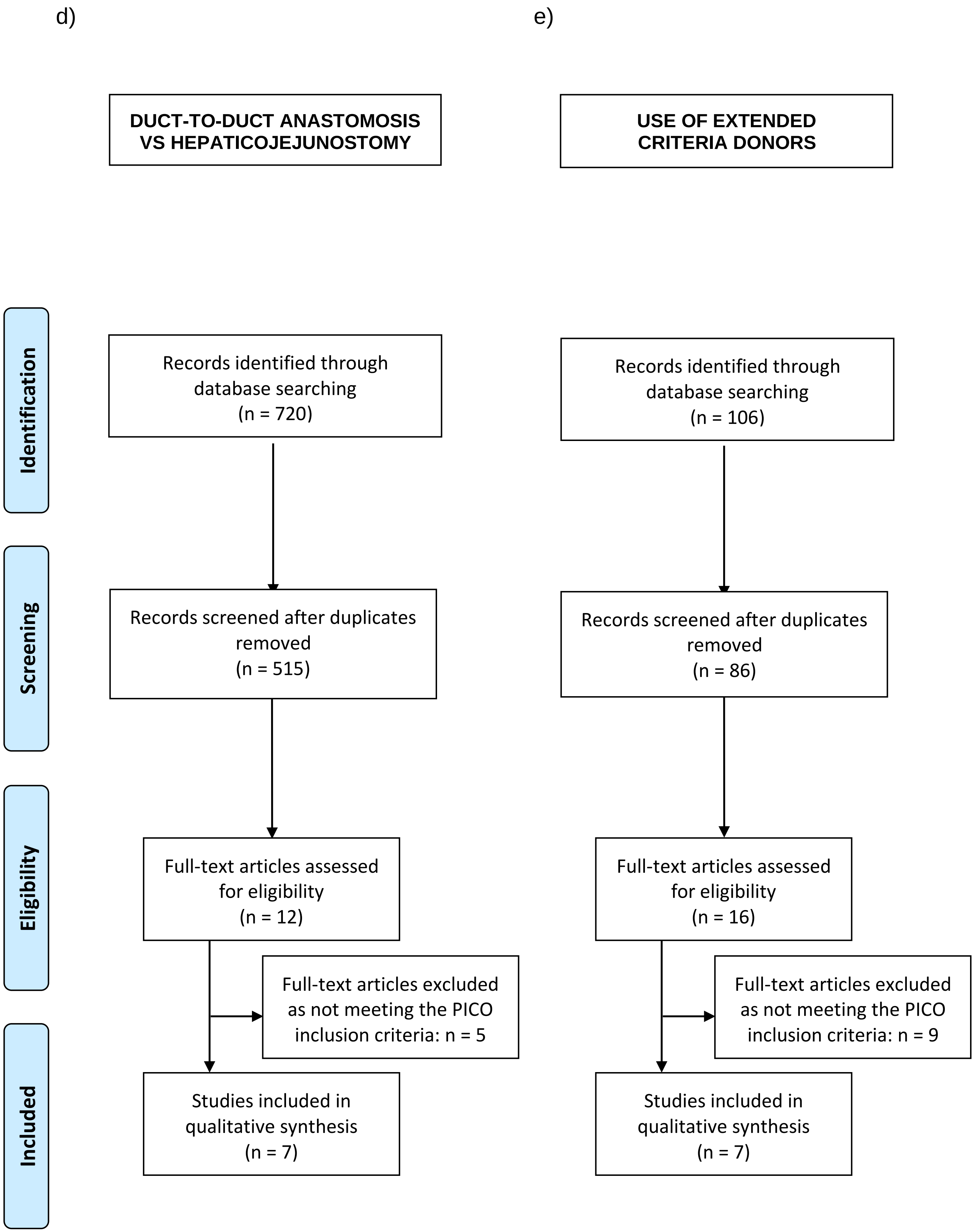
d)
DUCT-TO-DUCT ANASTOMOSIS VS HEPATICOJEJUNOSTOMY
Identification
Records identified through database searching (n = 720)
Screening
Records screened after duplicates removed (n = 515)
Eligibility
Full-text articles assessed for eligibility (n = 12)
Full-text articles excluded as not meeting the PICO inclusion criteria: n = 5
Included
Studies included in qualitative synthesis (n = 7)
e)
USE OF EXTENDED CRITERIA DONORS
Records identified through database searching (n = 106)
Records screened after duplicates removed (n = 86)
Full-text articles assessed for eligibility (n = 16)
Full-text articles excluded as not meeting the PICO inclusion criteria: n = 9
Studies included in qualitative synthesis (n = 7)

f)

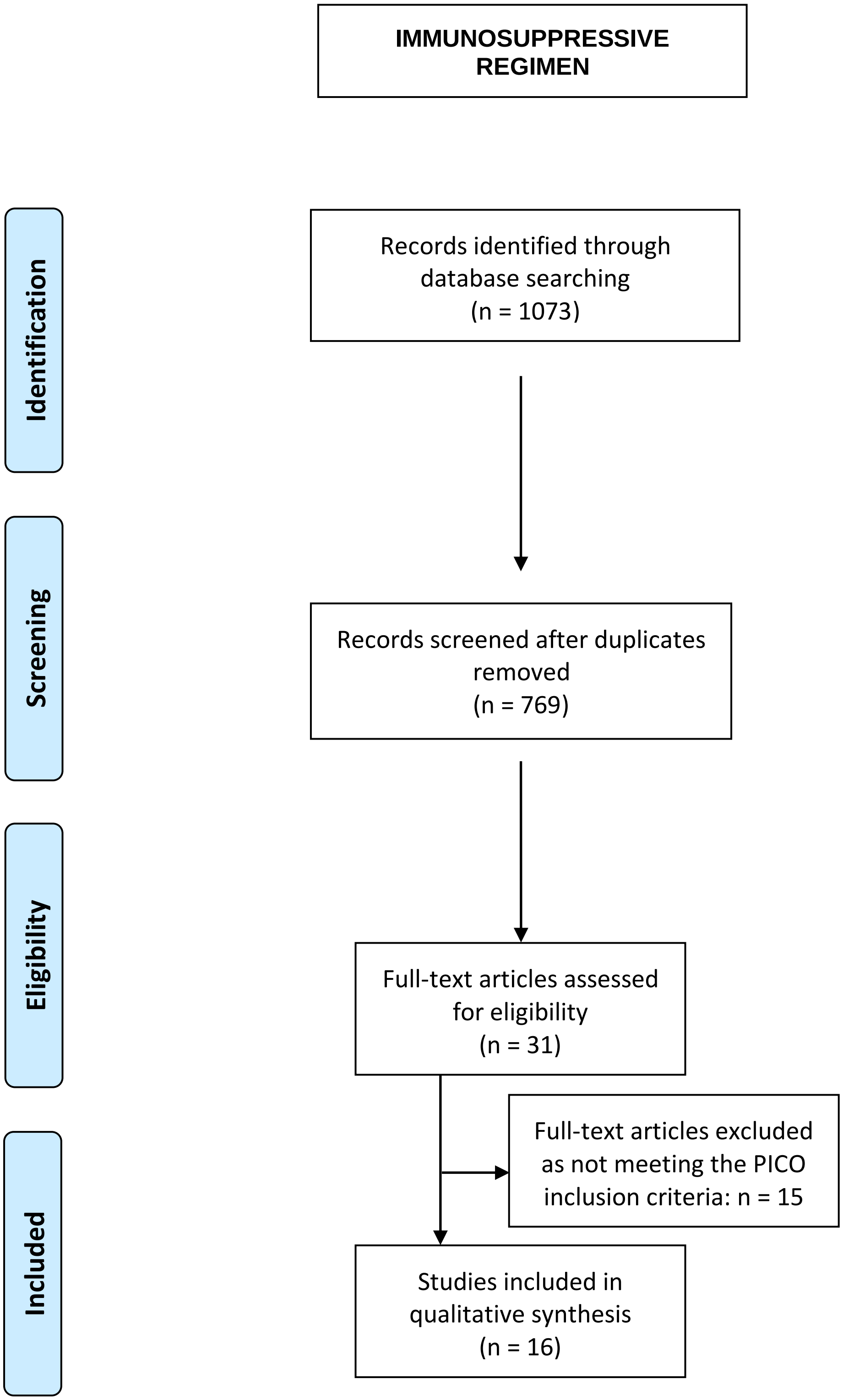
IMMUNOSUPPRESSIVE REGIMEN
Identification
Records identified through database searching (n = 1073)
Screening
Records screened after duplicates removed (n = 769)
Eligibility
Full-text articles assessed for eligibility (n = 31)
Full-text articles excluded as not meeting the PICO inclusion criteria: n = 15
Included
Studies included in qualitative synthesis (n = 16)

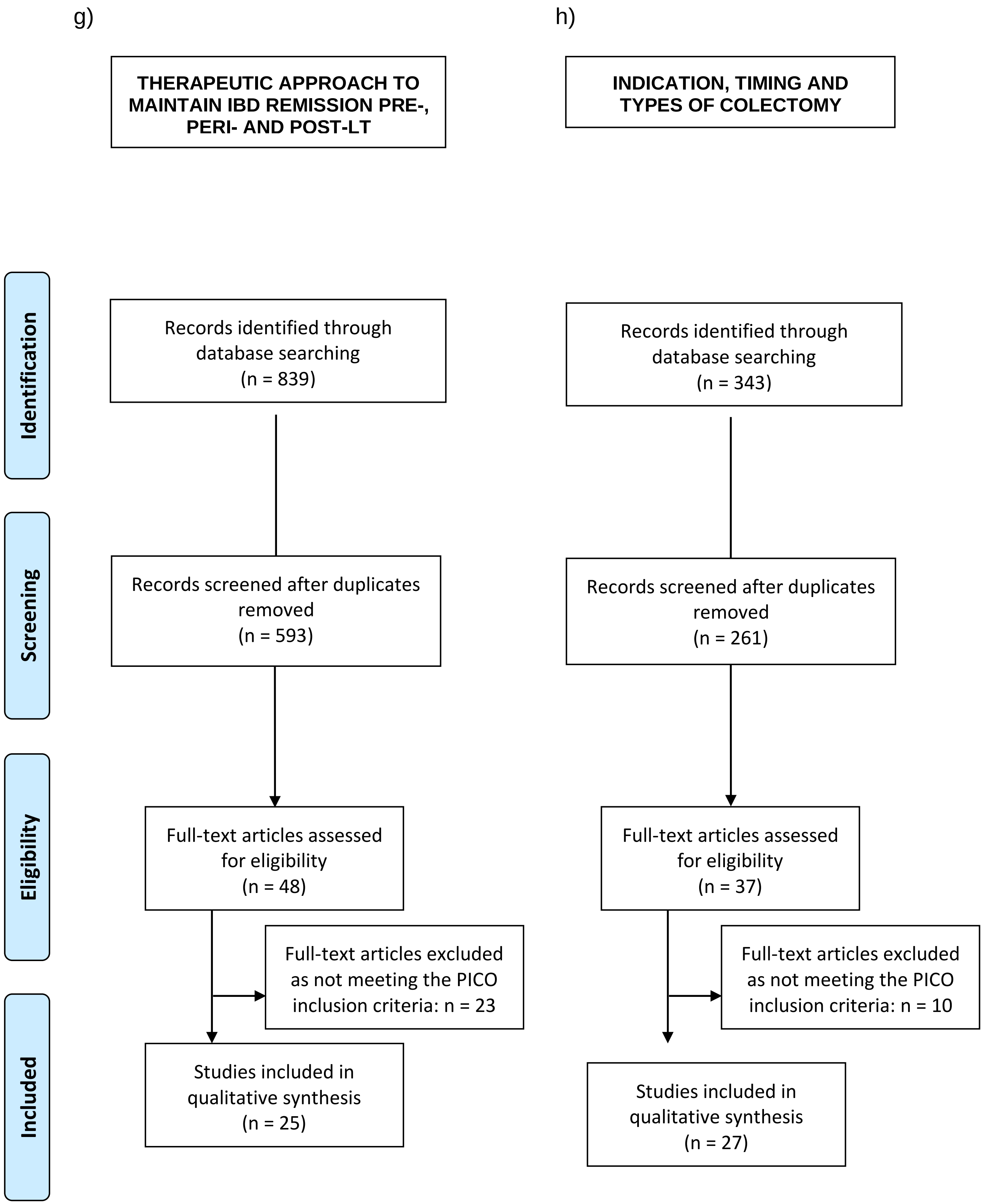
g)
THERAPEUTIC APPROACH TO MAINTAIN IBD REMISSION PRE-, PERI- AND POST-LT
Identification
Screening
Eligibility
Included
Records identified through database searching (n = 839)
Records screened after duplicates removed (n = 593)
Full-text articles assessed for eligibility (n = 48)
Full-text articles excluded as not meeting the PICO inclusion criteria: n = 23
Studies included in qualitative synthesis (n = 25)
h)
INDICATION, TIMING AND TYPES OF COLECTOMY
Records identified through database searching (n = 343)
Records screened after duplicates removed (n = 261)
Full-text articles assessed for eligibility (n = 37)
Full-text articles excluded as not meeting the PICO inclusion criteria: n = 10
Studies included in qualitative synthesis (n = 27)

i)

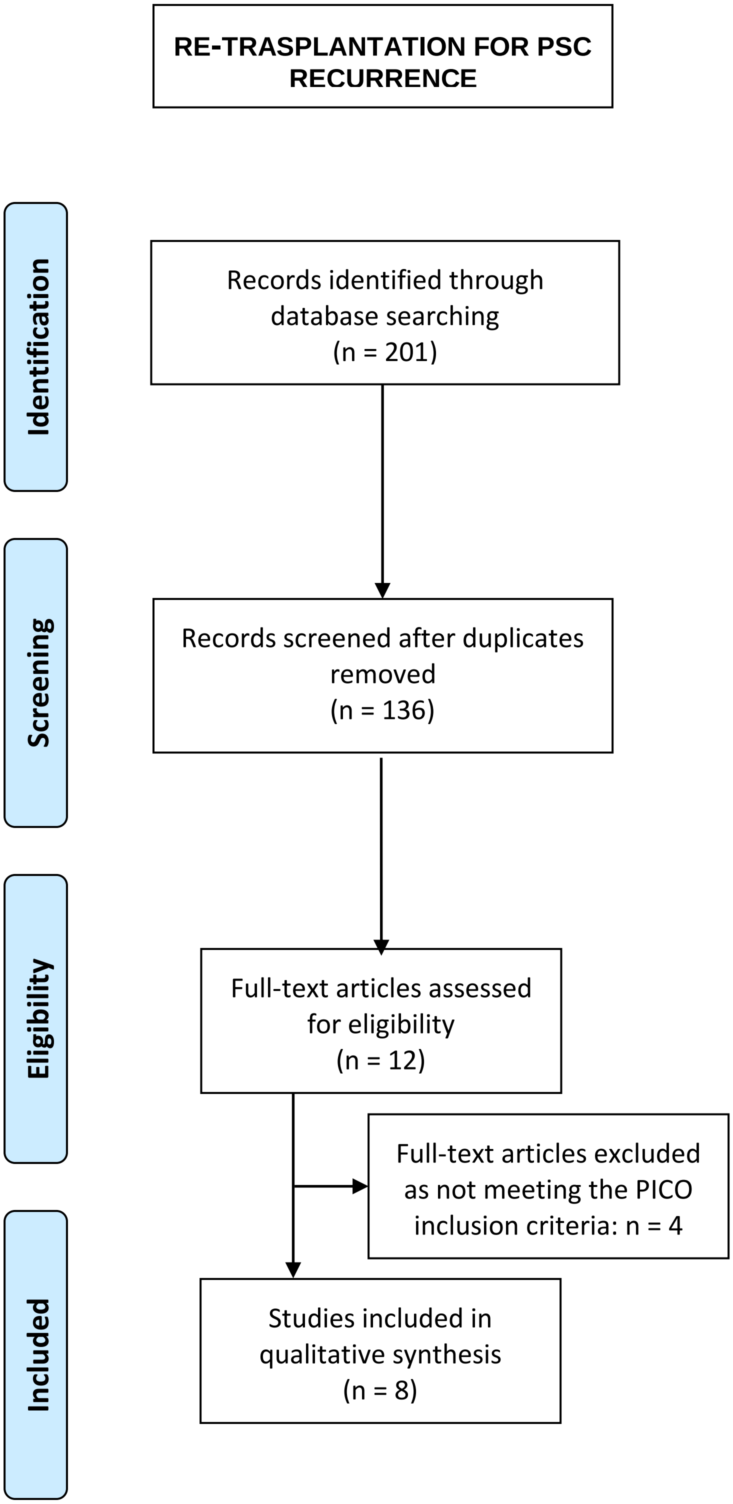
RE-TRASPLANTATION FOR PSC RECURRENCE
Identification
Screening
Eligibility
Included
Records identified through database searching (n = 201)
Records screened after duplicates removed (n = 136)
Full-text articles assessed for eligibility (n = 12)
Full-text articles excluded as not meeting the PICO inclusion criteria: n = 4
Studies included in qualitative synthesis (n = 8)

l)

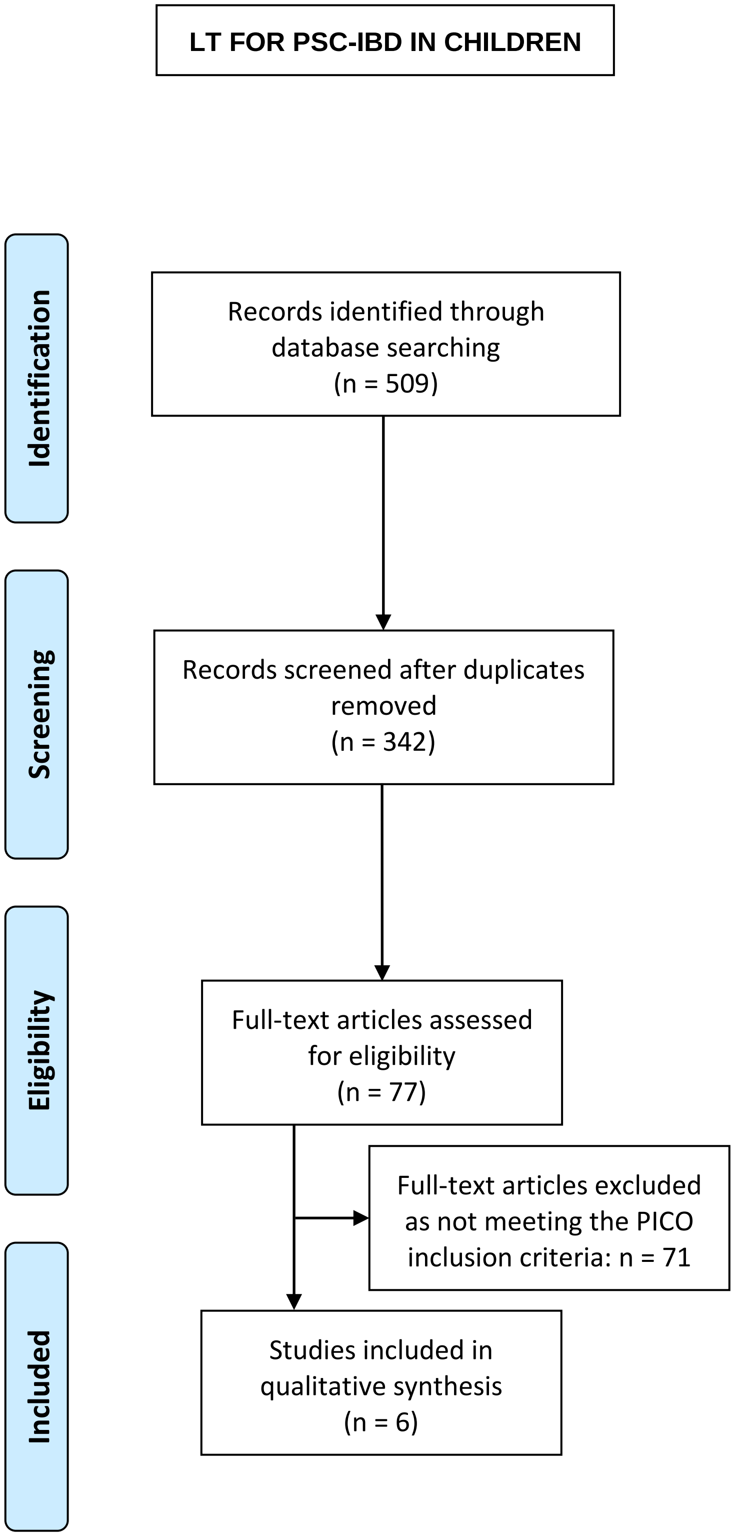
LT FOR PSC-IBD IN CHILDREN
Identification
Screening
Eligibility
Included
Records identified through database searching (n = 509)
Records screened after duplicates removed (n = 342)
Full-text articles assessed for eligibility (n = 77)
Full-text articles excluded as not meeting the PICO inclusion criteria: n = 71
Studies included in qualitative synthesis (n = 6)

Supplementary table 1. MELD allocation system in patients with PSC

Reference	Study type	Number of patients	Main outcomes
Nagai et al., Transpl Int 2021;34: 499-513.	Observational, retrospective	ALD=6094<br>HCV=1653<br>NASH=3848<br>PBC=602<br>PSC=819	- Disease progression<br>- Waitlist outcomes
Goet JC et al., Transpl Int. 2018 Jun;31(6):590-599	Observational, retrospective	Overall= 852<br>PSC=146	- Waitlist mortality<br>- Post-transplant survival
Klose et al., Langenbecks Arch Surg 2014;399: 1021–1029.	Observational, retrospective	Overall = 1420<br>PSC = 126	- Post-transplant survival in pre- and post-MELD era
Suri et al., J Clin Med. 2020 Jan 23;9(2):319	Observational, retrospective	AIH=7412<br>PBC= 8119<br>PSC=10901	- Waitlist survival (composite death or removal for clinical deterioration)
Goldberg et al., Liver Transpl. 2011 Nov;17(11):1355-63.	Observational, retrospective	Overall = 71976<br>PSC = 3165	- Times to death or withdrawal from the waitlist
Brandsaeter M et al., Liver Transpl. 2003 Sep;9(9):961-9	Observational, retrospective	PSC= 255<br>77% with IBD<br>Control = 610	- Events on the waitlist<br>- Events post-LT
Brandsaeter Scand J Gastroenterol. 2003 Nov;38(11):1176-83	Observational, retrospective	PSC=245<br>Control=618	- Post-LT survival
Goldberg et al., Liver Transpl. 2013, 19, 250–258	Observational, retrospective	PSC =171	- Waitlist survival (composite death or removal for clinical deterioration)

Abbreviations: ALD, alcohol-related liver disease; HCV, Hepatitis C virus; NASH, nonalcoholic steatohepatitis; PBC, primary biliary cholangitis; PSC, Primary Sclerosing Cholangitis; AIH, autoimmune hepatitis; IBD, Inflammatory Bowel Disease; LT, liver transplantation; MELD, model for end-stage liver disease; NA, not available.

Supplementary table 2. Pre-emptive LT for high-grade dysplasia in suspicious strictures in patients with PSC

Reference	Study type	Number of patients	Main outcomes
Andersen et al., Transpl Direct 2015;1:e39	Observational, retrospective	PSC=138<br>Suspicion of malignancy 25/138 (18.1%)<br>PBC+AIH=84	- Features of PSC LT recipients<br>- Post-LT outcomes
Majeed A et al., Scand J Gastroenterol 2018;53:56–63	Observational, retrospective	PSC=209<br>Benign lesions=169<br>CCA=16<br>HGD=12	- Post-LT survival
Boberg KM et al., J Hepatol 2006;45:568–574	Observational, retrospective	PSC=61<br>PSC with LGD or HGD/CCA=22<br>HGD LT=7	- Brush cytology vs final histopathology of the explanted liver
Vannas MJ et al., Liver Int 2017;37:735–742	Observational, retrospective	PSC=126<br>Symptomatic=96<br>Asymptomatic=35	- Brush cytology vs final histopathology of the explanted liver

Abbreviations: AIH, autoimmune hepatitis; CCA, cholangio-carcinoma; HGD, high grade dysplasia; LGD, low grade dysplasia; LT, liver transplantation; NA, not available; PBC, primary biliary cholangitis; PSC, Primary Sclerosing Cholangitis.

Supplementary table 3. Biliary stenting in patients on the waiting list

**Reference**	**Study type**	**Number of patients**	**Main outcomes**
Ponsioen CY et al., Gastroenterology. 2018;155(3):752-9	Prospective, randomized controlled study	PSC=65	- Cumulative recurrence-free patency of the primary dominant strictures<br>- Adverse events (cholangitis, pancreatitis)
Ferreira M et al., Clin Endosc. 2021;54(6):833-42	Systematic review Meta-analysis	PSC=467	- Clinical efficacy<br>- Transplant rates<br>- Adverse events
EASL Clinical Practice Guidelines on sclerosing cholangitis. J Hepatol. 2022;77(3):761-806.	Clinical practice guidelines European Association for the Study of the Liver	NA	NA
Aabakken L et al., Endoscopy. 2017;49(6):588-608.	Clinical practice guidelines European Society of Gastrointestinal Endoscopy (ESGE) and European Association for the Study of the Liver (EASL)	NA	NA

Abbreviations: NA, not available; PSC, Primary Sclerosing Cholangitis.

Supplementary table 4. Bacterial cholangitis in patients on the waiting list

**Reference**	**Study type**	**Number of patients**	**Main outcomes**
EASL Clinical Practice Guidelines on sclerosing cholangitis. J Hepatol. 2022;77(3):761-806.	Clinical practice guidelines	NA	NA
Rudolph et al., J Hepatol. 2009;51(1):149-55	Single-center prospective study on PSC patients	171	- A majority of patients with dominant stenosis had bacteria in bile<br>- Bacteria in bile had no effect on survival whereas Candida in bile was associated with reduced survival
Gomi et al., J Hepatobiliary Pancreat Sci. 2018;25(1):3-16.	Clinical practice guidelines for antimicrobial therapy for acute cholangitis/cholecystitis Based on a systematic literature review	NA	
Zigmond et al., Clin Gastroenterol Hepatol. 2022. Sep 16;S1542-3565(22)00879-5.	Single-centre retrospective study including PSC patients	189	- Bile fluid in PSC was frequently colonized with bacteria.<br>- Colonization of bile fluid with *Enterococcus sp.* was associated with PSC disease progression
Aabakken L et al., Endoscopy. 2017;49(6):588-608.	Clinical practice guidelines European Society of Gastrointestinal Endoscopy (ESGE) and European Association for the Study of the Liver (EASL)	NA	NA
Piano S et al., Gastroenterology. 2019;156(5):1368-80.	Multicenter retrospective study	1302	- The global prevalence of multidrug-resistant (MDR) bacteria was 34%<br>- The prevalence of MDR bacteria differed significantly among geographic areas

Abbreviations: MDR, multidrug resistance; NA, not available; PSC, Primary Sclerosing Cholangitis.

Supplementary table 5. Duct-to-duct anastomosis compared with hepaticojejunostomy

Reference	Study type	Number of patients	Main outcomes
Montano-Loza et al., Aliment Pharmacol Ther 2017, 45: 485-500.	Systematic review	NA	- Recurrence
Sutton, Liver Transplant. 2014;20:457–463.	Retrospective, observational	98 patients<br>45 duct-to-duct,<br>53 Roux-en-Y	- Patient and graft survival<br>- biliary leakage<br>- anastomotic strictures (AS)<br>- non-anastomotic strictures (NAS)<br>- cholangitis<br>- cholangiocarcinoma
Pandanaboyana, Transpl Int. 2015;28(4):485-91.	Systematic review and Meta-analysis	10 studies, 910 patients<br>338 duct-to-duct<br>572 Roux-en-Y	- Biliary strictures (AS/NAS)<br>- biliary leakage<br>- cholangitis<br>- cholangiocarcinoma
Wells, Transplant Proc. 2013 Jul-Aug;45(6):2263-71.	Systematic review and Meta-analysis	7 studies, 692 patients<br>245 duct-to-duct,<br>447 Roux-en-Y	- Patient and graft survival<br>- biliary leak<br>- disease recurrence<br>- biliary stricture
Al-Judaibi, Hepat Mon. 2015;15(5):e18811.	Retrospective observational	73 patients<br>15 duct-to-duct<br>58 Roux-en-Y	- Patient and graft survival<br>- biliary leak<br>- biliary stricture
Shamsaeefar, Clin Transplant. 2017;31(6).	Retrospective observational	405 patients<br>143 duct-to-duct<br>260 Roux-en-Y	- Patient and graft survival<br>- biliary leak<br>- stricture
Chazouilleres ,J Hepatol. 2022;77:761–806	EASL Clinical Practice Guidelines on sclerosing cholangitis	NA	

Abbreviations: AS, anastomotic strictures; NA, not available; NAS, non-anastomotic strictures; PSC, Primary Sclerosing Cholangitis.

Supplementary table 6. Graft selection in LT candidate with PSC

**Reference**	**Study type**	**Number of patients**	**Main outcomes**
Fleetwood et al. Exp Clin Transplant. 2021 Jun;19(6):563-569.	Retrospective observational study	95 patients,<br>28 DCD<br>67 DBD	- Graft failure<br>- graft survival<br>- patient survival
Kitajima, American Journal of Transplantation 2021;21(suppl 4):782	Retrospective observational study	3099 DBD<br>151 DCD	- Graft survival<br>- biliary complications<br>- PSC recurrence
Trivedi, J Hepatol. 2017 Nov;67(5):957-965.	Retrospective observational study	143 patients,<br>35 DCD<br>108 DBD	- Graft and patient survival<br>- vascular complications<br>- biliary strictures
Sundaram et al. Transplantation. 2015 May;99(5):973-978.	Retrospective registry study	1667 patients<br>75 DCD<br>1592 DBD	- Graft failure<br>- graft and patient survival<br>- biliary complications
Alabraba et al. Liver Transpl. 2009 Mar;15(3):330-340.	Retrospective observational study	263 patients<br>73 ECD<br>1592 Normal risk	- recurrent PSC
El-Ghazaly Harb et al. Hepatology 2010; 52(SUPPL):846A.	Retrospective observational study	148 patients	- Recurrent PSC (outcomes compared with respect to graft type and donor risk index [DRI])
Redfield et al. American Journal of Transplantation 2015;15(SUPPL):97.	Retrospective registry study	UNOS dataset<br>PSC and non-PSC LT stratified by DCD (3194) or DBD (103512). Exact group numbers not stated	- Graft survival<br>- biliary complications

Abbreviations: ECD, extended criteria donors; DCD, donor after cardiac death; DBD, donor after brain death; LT, liver transplantation; NA, not available; PSC, Primary Sclerosing Cholangitis.

Supplementary table 7. Immunosuppressive regimen

Reference	Study type	Number of patients	Main outcomes
Berenguer M et al., Transplantation. 2021 Oct 1;105(10):2255-2262.	Registry analysis of ELTR	6463 patients with PSC	- survival
Irlès-Depé M et al., Liver Transpl. 2020 Nov;26(11):1477-1491.	Retrospective analysis of 4 French transplant centers	87 PSC (52 with IBD)	- survival
Peverelle, M et al., Liver Transpl., 27: 770-771.	Retrospective analysis from 2 centers	112 patients with PSC	- Recurrent PSC<br>- Colorectal cancer/high-grade dysplasia
Steenstraten IC et al., Aliment Pharmacol Ther. 2019 Mar;49(6):636-643.	Meta-analysis until 2018	2159 patients	- Recurrent PSC
Chen C et al., Medicine (Baltimore). 2020 May;99(20):e20205.	Meta-analysis until 2019	5077 with autoimmune liver disease	- Recurrent PSC
Pellegrin S. et al., Transplantation 2019	Retrospective study	57 PSC	- Recurrent PSC
Lindström L et al., Scand J Gastroenterol. 2018 Mar;53(3):297-304.	Retrospective analysis Nordic Transplant Registry	440 patients with PSC	- Recurrent PSC
Mouchli MA et al., Inflamm Bowel Dis. 2018 Apr 23;24(5):1074-1081	Retrospective single center study	373 patients with PSC	- Course post-LT
Jørgensen KK et al., Clin Gastroenterol Hepatol. 2013 May;11(5):517-23	Longitudinal study from Nordic Liver Transplant Group	439 patients with PSC	- IBD activity after transplantation
Singh S et al., Am J Gastroenterol. 2013 Sep;108(9):1417-25	Review	NA	- IBD activity after transplantation
Visseren T et al., JHEP Rep. 2022 Oct 1;4(12):100599	International retrospective study	531 patients with PSC	- Recurrent PSC
Filipec Kanizaj T et al., World J Gastroenterol. 2017 May 14;23(18):3214-3227	Review	NA	- IBD activity after transplantation

Joshi D et al., Liver Int. 2013 Jan;33(1):53-61.	Retrospective study	110 patients	- Graft failure
Liu K et al., Expert Rev Gastroenterol Hepatol. 2017 Oct;11(10):949-960	Expert review	NA	NA
Montano-Loza, A.J. et al., Aliment Pharmacol Ther 2017, 45: 485-500	Systematic review	NA	- Recurrent PSC

Abbreviations: IBD, inflammatory bowel disease; LT, liver transplantation; NA, not available; PSC, Primary Sclerosing Cholangitis.

Supplementary table 8. Management of IBD before and after liver transplant for PSC

**Reference**	**Study type**	**Number of patients**	**Main outcomes**
Mouchli, Inflamm Bowel Dis. 2020;26(12):1901-8.	Retrospective cohort study	373 PSC patients who had LT (total)<br>151 PSC-IBD patients	- IBD course post-LT<br>- rates of de novo IBD<br>- immunosuppression for IBD post-LT
Singh, Am J Gastroenterol. 2013;108(9):1417-25.	Review article	NA	Inflammatory bowel disease after liver transplantation for primary sclerosing cholangitis
Jorgensen, Clin Gastroenterol Hepatol. 2013;11(5):517-23.	Longitudinal cohort study	439 PSC patients who had LT (total)<br>218 PSC patients with intact colons	-IBD course post-LT<br>- immunosuppression for IBD post-LT
Chazouilleres ,J Hepatol. 2022;77:761–806.	EASL Clinical Practice Guidelines on sclerosing cholangitis	NA	NA
ESGE/EASL, J Hepatol. 2017;66(6):1265-81.	ESGE/EASL clinical guidelines on the role of endoscopy in primary sclerosing cholangitis	NA	NA
Lightner, Gastroenterol Rep (Oxf). 2017;5(3):165-77.	Review article	NA	- Preoperative, perioperative and postoperative immunosuppression for Crohn's disease
Aberra, Gastroenterology. 2003;125(2):320-7.	Retrospective cohort study	159 IBD patients having elective bowel surgery (total)<br>56 steroids<br>52 thiopurines<br>51neither	- Postoperative infection risk
Colombel, Am J Gastroenterol. 2004;99(5):878-83.	Retrospective cohort study	270 Crohn's disease patients (total)<br>107 steroids<br>105 thiopurines/ methotrexate<br>52 infliximab	- Early post-operative complications
Spadaccini, United European Gastroenterol J. 2019;7(7):875-80.	Systematic review	8 studies, 31 patients	- Vedolizumab outcomes in transplant recipients

Mohabbat, Aliment Pharmacol Ther. 2012;36(6):569-74.	Retrospective cohort study	8 IBD patients who had LT	- Efficacy and safety of anti-TNF agents
Altwegg, Dig Liver Dis. 2018;50(7):668-74.	Retrospective cohort study	18 PSC-IBD patients who had LT	- Efficacy and safety of anti-TNF agents
Shaikh, Pharmacotherapy. 2017;37(12):1578-85.	Review article	NA	- Safety and efficacy of biologic agents for the management of IBD after LT
Montano-Loza, Aliment Pharmacol Ther. 2017;45(4):485-500.	Systematic review	13 studies, 2595 patients (total)<br>AIH 122<br>PBC 531<br>PSC 1930<br>Overlap syndrome 12	- Recurrent autoimmune liver disease post-LT
Trivedi, Gastroenterology. 2020;159(3):915-28<br>.	Retrospective cohort study	2588 PSC-IBD patients	- Effects of PSC on IBD and HPB disease
Boonstra, Hepatology. 2013;58(6):2045-55.	Retrospective cohort study	590 PSC patients	- PSC outcomes
Claessen, J Hepatol. 2009;50(1):158-64.	Retrospective cohort study	211 PSC patients	- Risk of CCA and CRC in PSC patients<br>- survival
El-Matary, Clin Gastroenterol Hepatol. 2021;19(5):1067-70 e2.	Retrospective cohort study	509 paediatric PSC-IBD patients	- Incidence of CRC in paediatric PSC-IBD
Trivedi, Gut. 2021;70(10):1989-2003.	Review article	NA	Recent advances in clinical practice – epidemiology of AI liver disease
Krugliak, Clin Gastroenterol Hepatol. 2018;16(1):68-74.	Retrospective cohort study	143 PSC-IBD patients	- Incidence of subclinical IBD in PSC
Claessen, Inflamm Bowel Dis. 2009;15(9):1331-6.	Retrospective cohort study	27 IBD-CRC<br>127 PSC-IBD-CRC	- Clinical course of IBD-CRC in patients with and without PSC
Vera, Transplantation. 2003;75(12):1983-8.	Retrospective cohort study	152 PSC patients (total)<br>100 PSC-IBD	- Risk factors for CRC in PSC patients post-LT
Higashi, Hepatology. 1990;11(3):477-80.	Case reports	2 PSC-IBD patients	- PSC-IBD outcomes post-LT

Venkatesh, J Crohns Colitis. 2013;7(12):968-73.	Retrospective cohort study	10 PSC-IBD patients	- Clinical course of LGD in PSC-IBD
van Schaik, Nat Rev Gastroenterol Hepatol. 2009;6(11):671-8.	Review articles	NA	- Endoscopic and pathological aspects of colitis-associated dysplasia
Lamb, Gut. 2019;68(Suppl 3):s1-s106.	BSG consensus guidelines on the management of inflammatory bowel disease in adults	NA	NA
Brown, Colorectal Dis. 2018;20 Suppl 8:3-117.	ACPGBI consensus guidelines in surgery for IBD	NA	NA
Poritz, Dis Colon Rectum. 2003;46(2):173-8.	Retrospective cohort study	16 PSC-IBD patients	- Outcomes of surgical management of IBD in PSC
Cho, J Gastrointest Surg. 2008;12(7):1221-6.	Retrospective cohort study	22 PSC-IBD patients	- Outcomes of IPAA post-LT for PSC-IBD
Navaneethan, Gastroenterol Rep (Oxf). 2016;4(1):43-9.	Retrospective cohort study	273 PSC-IBD patients (total)<br>223 UC<br>50 CD	- Comparative outcomes of PSC-UC and PSC-CD
Roberts, BMJ. 2007;335(7628):1033.	Retrospective cohort study	23464 IBD patients<br>5480 colectomies for IBD	- Mortality outcomes after colectomy for IBD
Dong, Aliment Pharmacol Ther. 2020;51(1):8-33.	Systematic review and meta-analysis	53 studies, 744299 patients with ASUC	- Mortality outcomes of ASUC
Mahmud, Hepatology. 2021;73(1):204-18.	Retrospective cohort study	3785 cirrhotic patients	- Surgical risk model for cirrhosis
Mahmud, Clin Gastroenterol Hepatol. 2022;20(5):e1121-e34.	Retrospective cohort study	4712 surgeries in cirrhotic patients	- Risk of postoperative complications in cirrhotic patients
Navaneethan, Clin Gastroenterol Hepatol. 2012;10(5):540-6.	Retrospective cohort study	167 PSC-IBD patients	- Impact of PSC severity on IBD outcomes (colectomy)
Marelli, Gut. 2011;60(9):1224-8.	Retrospective cohort study	96 PSC-IBD patients	- Impact of PSC severity on IBD outcomes<br>- outcomes transplanted

			vs non-transplanted PSC-IBD
Navaneethan, Aliment Pharmacol Ther. 2012;35(9):1045-53.	Retrospective cohort study	222 PSC patients (total)<br>167 PSC-IBD<br>55 PSC-IBD	- Impact of IBD on PSC outcomes
Ong, Gastroenterol Hepatol Bed Bench. 2018;11(4):277-83.	Systematic review and meta-analysis	7 studies,<br>167 PSC patients with colectomy,<br>186 PSC patients without colectomy	- Impact of gut-liver axis on PSC activity
Cangemi, Gastroenterology. 1989;96(3):790-4.	Prospective cohort study	45 PSC IBD patients (total)<br>20 colectomy<br>25 no colectomy	- Impact of colectomy on PSC
Nordenvall, Aliment Pharmacol Ther. 2018;47(2):238-45.	Retrospective cohort study	2594 PSC-IBD patients	- Impact of colectomy on PSC outcomes
Chapman, Gut. 1980;21(10):870-7.	Review article	NA	- Primary sclerosing cholangitis: a review of its clinical features, cholangiography, and hepatic histology
Thompson, Ann Surg. 1982;196(2):127-36.	Retrospective cohort study	37 PSC patients	- Clinicopathological features of PSC cases
Martin, Ann Surg. 1990;212(4):551-6; discussion 6-8.	Retrospective cohort study	178 PSC patients	- Surgical considerations in PSC
Sathiaseelan, J Paediatr Child Health. 2022;58(7):1221-7.	Retrospective cohort study	51 paediatric PSC patients	- Impact of IBD on liver-related outcomes in paediatric PSC
Kochhar, Gastroenterol Rep (Oxf). 2015;3(3):228-33.	Retrospective, case-control study	9 PSC-IBD with TIPS pre-colectomy<br>37 PSC-IBD with colectomy only	- Impact of TIPS on post-colectomy outcomes in PSC-IBD patients
Alabraba, Liver Transpl. 2009 Mar;15(3):330-340.	Prospective cohort study	230 PSC patients who had LT<br>162 PSC IBD	- Impact of colectomy on recurrent PSC
Nordenvall, Inflamm Bowel Dis. 2018;24(3):624-32.	Retrospective cohort study	49882 UC patients,<br>2079 PSC-UC patients	- Outcomes of surgical treatment of UC with or without PSC
Pavlides, J Crohns Colitis. 2014;8(7):662-70.	Retrospective cohort study	21 PSC-IBD-IPAA<br>79 IBD-IPAA	- Outcomes of IPAA in PSC
Block, J Crohns Colitis. 2014;8(5):421-30.	Retrospective case-control study	48 PSC-UC<br>31 IPAA<br>17 IRA	- Outcomes of IPAA and IRA in PSC-UC

Ponsioen, Gastroenterology. 2018;155(3):752-9 e5.	Randomized-controlled trial	31 PSC patients<br>31 balloon dilatation<br>34 short term stent	- Efficacy and safety of balloon dilatation vs short term stents in PSC for dominant strictures
Wiesner, Gastroenterology. 1986;90(2):316-22.	Randomized-controlled trial	70 PSC patients (total)<br>19 proctocolectomy and ileostomy<br>4 proctocolectomy and ileoanal anastomosis	- Risk of parastomal varices following surgical resection
Trivedi, Aliment Pharmacol Ther. 2018;48(3):322-32.	Retrospective cohort study	75 PSC-IBD who had colectomy<br>21 IPAA<br>54 end ileostomy	- Impact of IPAA and end ileostomy on post-LT liver outcomes
Mol, UEG Journal 2022;10:LB02.	Retrospective cohort study	1341 PSC patients (total)<br>912 PSC-IBD<br>187 PSC-IBD had colectomy	- Impact of colectomy on PSC disease course

Abbreviations: NA, not available; PSC, Primary Sclerosing Cholangitis; IBD, inflammatory bowel disease; LT, liver transplantation; TNF, tumor necrosis factor; AIH, autoimmune hepatitis; PBC, primary biliary cirrhosis; CCA, cholangio-carcinoma; CRC, colorectal cancer; LGD, low-grade dysplasia; IPAA, ileal pouch-anal anastomosis; ASUC, acute severe ulcerative colitis; TIPS, transjugular intrahepatic portosystemic shunt; IRA, ileorectal anastomosis.

Supplementary table 9. Assessment of criteria of futility for re-LT in case of rPSC

**Reference**	**Study type**	**Number of patients**	**Main outcomes**
Egawa H et al. Am J Transplant. 2011;11(3):518-27.	Retrospective cohort study	114 patients undergoing primary LDLT	- Long-term patient and graft survival
Moncrief KJ et al. Can J Gastroenterol. 2010;24(1):40-6.	Retrospective cohort study	59 (71% with IBD pre-LT)	- Patients survival
Campsen J et al. Liver Transpl. 2008;14(2):181-5.	Retrospective cohort study	130 PSC patients who had LT	- PSC recurrence<br>- Retransplant<br>- Patient survival
Goss JA et al. Ann Surg. 1997;225(5):472-81; discussion 81-3.	Retrospective cohort study	127 PSC patients who had LT	- PSC recurrence<br>- Cholangiocarcinoma<br>- Retransplant<br>- Patient and graft survival
Ravikumar R et al. J Hepatol. 2015;63(5):1139-46.	Retrospective cohort study	679 PSC patients who had LT (61% with IBD)	- PSC recurrence<br>- Retransplant<br>- Patient survival
Lindstrom L et al. Scand J Gastroenterol. 2018;53(3):297-304.	Analysis of the Nordic Liver Transplant Registry	440 PSC patients who had LT	- PSC recurrence<br>- Retransplant<br>- Patient survival
Neuberger J et al. Lancet. 1999;354(9190):1636-9.	Review	NA	- Guidelines for selection of patients for liver transplantation in the era of donor-organ shortage
Henson J et al. Liver Transpl. 2017;23(6):769-80.	Analysis of the United Network for Organ Sharing/Organ Procurement and Transplantation Network database	5080 PSC patients who had LT	- PSC recurrence<br>- Retransplant<br>- Patient survival

Abbreviations: IBD, inflammatory bowel disease; LT, liver transplantation; NA, not available; PSC, Primary Sclerosing Cholangitis; rPSC, recurrent primary sclerosing cholangitis.
